# Digital Tools to Support the Systematic Review Process: An Introduction

**DOI:** 10.1111/jep.70100

**Published:** 2025-04-28

**Authors:** Lena Schmidt, Ian Cree, Fiona Campbell, WCT EVI MAP group

**Affiliations:** ^1^ National Institute for Health and Care Research Innovation Observatory, Population Health Sciences Institute Newcastle University Newcastle upon Tyne UK; ^2^ International Agency for Research on Cancer Lyon France

**Keywords:** artificial intelligence, automation tools, machine‐learning, pathology, systematic review automation

## Abstract

**Background:**

The introduction of systematic reviews in medicine has prompted a paradigm shift in employing evidence for decision‐making across various fields. Its methodology involves structured comparisons, critical appraisals, and pooled data analysis to inform decision‐making. The process itself is resource‐intensive and time‐consuming which can impede the timely incorporation of the latest evidence into clinical practice.

**Aim:**

This article introduces digital tools designed to enhance systematic review processes, emphasizing their functionality, availability, and independent validation in peer‐reviewed literature.

**Methods:**

We discuss digital evidence synthesis tools for systematic reviews, identifying tools for all review processes, tools for search strategy development, reference management, study selection, data extraction, and critical appraisal. Emphasis is on validated, functional tools with independently published method evaluations.

**Results:**

Tools like EPPI‐Reviewer, Covidence, DistillerSR, and JBI‐SUMARI provide comprehensive support for systematic reviews. Additional tools cater to evidence search (e.g., PubMed PICO, Trialstreamer), reference management (e.g., Mendeley), prioritization in study selection (e.g., Abstrackr, EPPI‐Reviewer, SWIFT‐ActiveScreener), and risk bias assessment (e.g., RobotReviewer). Machine learning and AI integration facilitate workflow efficiency but require end‐user informed evaluation for their adoption.

**Conclusion:**

The development of digital tools, particularly those incorporating AI, represents a significant advancement in systematic review methodology. These tools not only support the systematic review process but also have the potential to improve the timeliness and quality of evidence available for decision‐making. The findings are relevant to clinicians, researchers, and those involved in the production or support of systematic reviews, with broader applicability to other research methods.

## 1 Background

In 1973, Archie Cochrane, a physician and epidemiologist, wrote ‘Effectiveness and Efficiency’ [1], prompting what many have called a paradigm shift within medicine and one which has rippled out across a diverse range of disciplines from education, social care, policing and environmental sciences. So profound has been the shift in thinking that the use of evidence to support decisions is now part of our normal policy and practice discourse.

The scientific methodology used to bring the best evidence to the heart of decision making is called systematic review. In 1993 the Cochrane library was established, creating the first database of systematic reviews to support decision making in healthcare, to provide evidence to lead to inform best use of limited resources, improve healthcare, and ultimately save lives. One of Cochrane's first milestone reviews showed that not only was there a significant lag in practice taking up clear evidence of effectiveness, but that research continued to be undertaken long after a pooling of the existing data would have shown a clear benefit. Systematic reviews offer the potential to reduce waste, as well as save lives. Cochrane, other organisations’ and initiatives as such as the Campbell Collaboration, JBI, GRADE, PRISMA have also pioneered the methodology, ensuring that systematic reviews were rigorous, free from bias, transparent in their methods and ultimately trustworthy. Systematic reviews make sense of the science by enabling structured comparison, critical appraisal and where primary studies are sufficiently similar, pooled for an overall effect of an intervention and potentially of their harms. The methodology justifies the place of systematic reviews as the most trusted form of evidence to inform decision making, at the top of the pyramid of evidence.

These developments have led to an exponential growth both in the demand and in the publication of systematic reviews. However, the classic systematic review approach is not without its critics. To be useful, evidence must be produced in a timely manner, be up to date, be contextually relevant, address questions beyond just what works, but also for whom, in w hat circumstances [2]. The process of undertaking a systematic review is costly, requiring approximately a year for a team to complete if the recommended approaches are applied. Failure to do so risks producing a review which can't be trusted and ultimately putting lives potentially at risk. We know that the policy making time frames and contexts often make existing reviews unhelpful. Moreover, the unprecedented growth of the biomedical literature has increased the burden on those trying to make sense of the published evidence base [3]. To support reviewers in conducting more timely evidence‐synthesis, Cochrane, JBI, and Campbell also support the development of digital evidence synthesis tools and to educate reviewers about their responsible usage—to support a new generation of software and infrastructure that can help expedite systematic reviews by integrating digital management of workflows and automation through artificial intelligence in a sustainable and safe manner.^1^

The need to improve the efficiency of the systematic process has long been a goal for those undertaking systematic reviews to support decision making, and has led to the development of ‘rapid review methods,’ which incorporate methodological shortcuts, but also efficiencies that enable reviews to be undertaken within shorter timeframes [4]. The need for greater efficiency in the review process, but also the requirements to work across a team, on large datasets has led to the growth of tools available to support those undertaking reviews and improve the workflow, management and team working of the systematic review.

Systematic review methods have also evolved, this can be seen for example via the evolution of the Cochrane Handbook for Systematic Reviews of Interventions publishing 10 updated versions since 2003,^2^ and the methods applied to a wider diversity of research questions beyond simply the question ‘Is it effective’. Systematic review methods have been applied to address questions that seek to understand why something works, in which population. The terminology has evolved to reflect some of those changes, and the term ‘evidence synthesis’ now provides a useful umbrella term under which the wide variety of types of systematic review can sit. However, methodological advances that enable synthesis of evidence beyond randomised controlled trials remain underutilised. Two important review types are diagnostic accuracy and prognosis. However, there exists a lack of diagnostic and prognostic reviews, which expresses itself for example in their coverage within the Cochrane library. As of December 2024, there existed 8966 published interventional reviews, but only 192 diagnostic and 21 prognostic reviews. Many health conditions could benefit from a better coverage in diagnostic and prognosis review types; especially pathology where plenty of not‐yet‐synthesised primary literature exists. Supporting the early diagnosis of cancers is as crucial as understanding the prognosis of the disease with respect to specific personal factors, such as gene expressions and their interplay with the effectiveness of a treatment and patient survival. However, robust and reliable digital tools are needed to support reviewers in conducting these complex and oftentimes large review projects.

**Figure 1:**
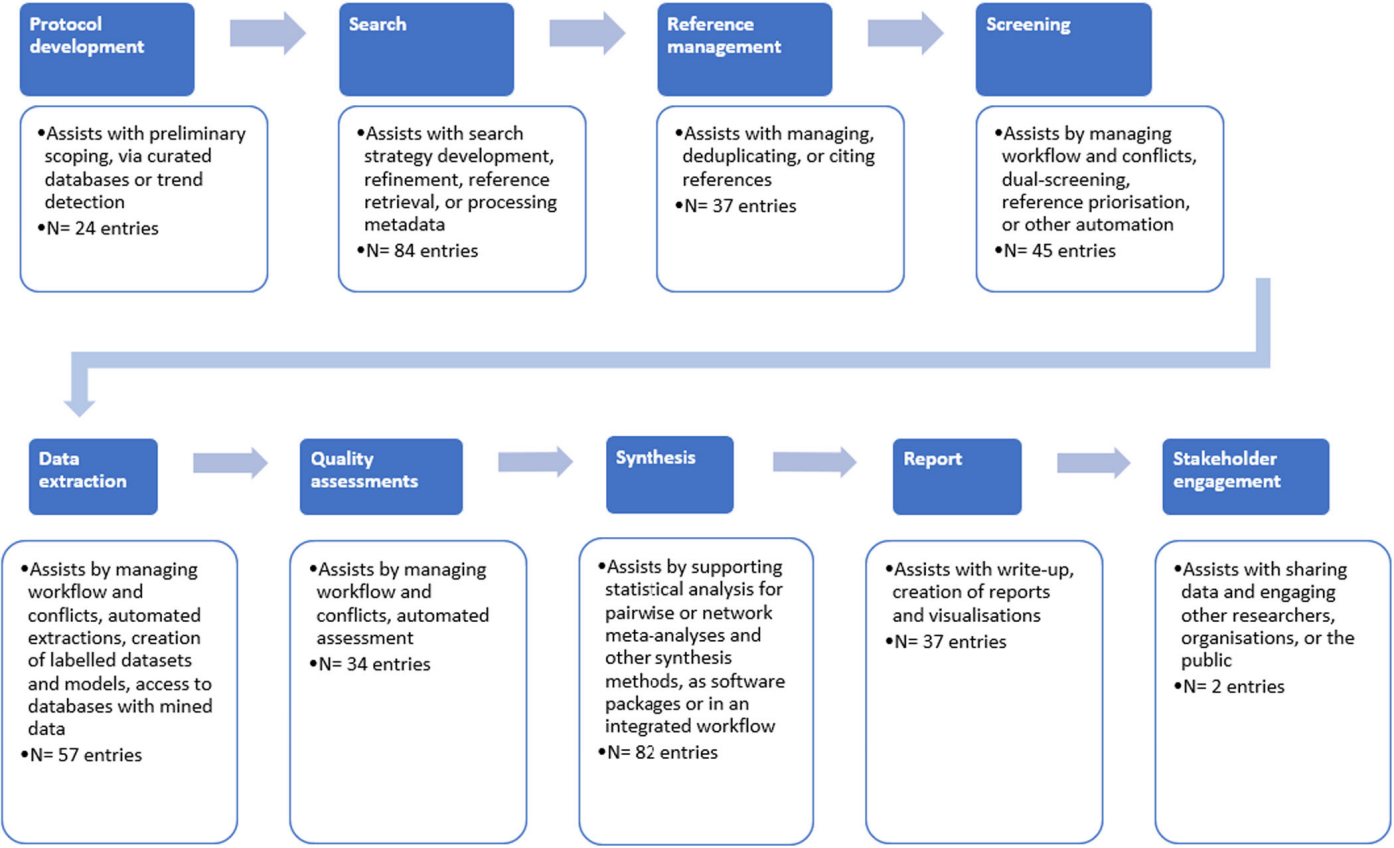
Software packages and tools listed in the ‘Systematic Review Toolbox’ website (available at https://systematicreviewtools.com/). As of 31/12/2024, it listed 235 unique entries for tools and software packages to support any type of literature review across nine steps in the review workflow.

For anyone embarking on a systematic review, there are a range of courses, both face to face, on‐line as well as published material that can support you as you apply the methods to your review question. An array of different digital tools are available that can support the review process (see Figure 1) [5]. We define ‘digital tools’ as software applications that provide a digital environment for conducting reviews, for example by uploading and managing references, managing screening and data extraction processes, or providing user interfaces to point out and resolve conflicting answers between reviewers. Oftentimes, these tools are deployed online, which creates a collaborative environment for reviewers to manage, divide and conquer workload. A positive side‐effect of deployment online is the possibility of integrating artificial intelligence into the workflow within a tool. Despite the availability of a growing number of such tools, we know that most commonly, digital collaborative tools are used for reference screening [6], while data extraction or risk of bias assessment (due to the potential complexity or variability of data) is often carried out with the help of customisable spreadsheets within widely available tools such as Excel or Google Docs. Other frequently used tools are Word for write‐up and a refence management software to manage search results, such as Mendeley.^3^

If used correctly, digital tools offer reviewers considerable potential to undertake their review with greater efficiency and therefore enable time to be invested in the critical appraisal, synthesis, interpretation and communication of the findings [6].

## 2 Aim

This paper aims to provide a brief introduction to a selection of the tools available, what aspects of the review they can support, their availability and published validations. We selected tools based on the scope of their functionality and availability of published methods and independent validations. Due to the large amount of existing, and sometimes non‐maintained or dysfunctional tools^4^ we are not presenting a systematic presentation of all tools. The range of tools available and the increasing range of tools using Artificial Intelligence is evolving, this paper will highlight where advances are currently being made and what the future may hold.

Any systematic review will need the following requirements, and digital tool usage should not infringe or reduce any of these underlying paradigms:

− Methods that are reproducible and transparent. This means that good record keeping will be necessary so that you can report all of the methods used and findings. Journals will require that reviews are PRISMA compliant [7]. It is useful to look at the PRISMA statement before beginning your protocol because automation approaches such as reducing the amount of records to screen are covered, giving a good sense of what you will need to report and hence need to record.
− A team; usually including an information specialist, two or more reviewers, a topic expert and a knowledge user. At least one member of the team should additionally possess methodological systematic review knowledge. Therefore, there need to be ways to share information. Digital tools should enable these types of workflows and teamwork, for example by promoting independent dual screening or facilitating the process of resolving disagreements in dual data extraction and bias assessment across different members of the team.
− Reviews have components with different tool requirements: time management, storing references, screening references, data extraction, bias assessment and assessing certainty, synthesis and finally report writing. When using multiple tools, data transfer between them needs to be carried out with care, to avoid losing data.

## 3 The ‘Big Four’ or the One‐Stop‐Shop Tools

Four tools exist that support the entire systematic review process (for interventional reviews) including managing the search results, facilitating the process of screening, enabling data extraction, bias assessment, supporting the process of synthesis and also visualising the results (see Table 1). While it is possible to use them for diagnostic or prognosis reviews, it is important to keep in mind that design and validation of automation methods may have been focused on evidence from randomised trials and that the tool infrastructure might require further manual customisation and implementation of bias assessment tools or complex data extraction forms. However, most importantly these tools also support team‐working and enable independent dual screening and data extraction which is a basic requirement for all review types. For each tool there is training and familiarisation required. EPPI‐reviewer, for example has a responsive help desk and a suite of on‐line support material.

**Table 1:** Tools to support the entire systematic review process.

Tool	Subscription model
EPPI‐Reviewer	Monthly (per review, per user)^a^. Code is open‐source
Covidence	Annual (individual, package, organisational)^a^
DistillerSR	Monthly or annual
JBI‐SUMARI	Annual (individual, organisational)

a Free to authors of Cochrane and Campbell reviews.

There are free alternative tools which also offer functions to support parts of the review process including deduplication, screening, sharing and re‐using data, extracting data and more:

ASReview Lab, CADIMA, SysRev, SRDR+, SR‐Accelerator, HubMeta.

## 4 Tools to Support Question Development and Locating the Evidence

There are a number of tools that can support the process of searching for relevant evidence. They may also provide crude overviews of the numbers of expected references—thus supplying information that supports research question development and refinement as well as the planning of a review process. They include the PubMed PICO (Patient, Intervention, Comparison, Outcome) tool from the National Library of Medicine which is a research instrument that can aid the identification of literature on a variety of health related topics. It can give a sense of the scale of the literature and key terms that will be built into a full search strategy but would not replace a search strategy that would be developed to be used in other databases [8]. Another PubMed tool is the PubReMiner,^5^ which also supports building a search strategy. It allows you to enter relevant references, and it then determines the high frequency words, MeSH terms and subheadings that should be added to a search strategy locating evidence of the type submitted.

Other tools or software packages that can support the development of search strategies include Yale MeSH analyser,^6^ WordFreq^7^ and litsearchr^8^ all of which can support the development of search strategies by identifying important terms to include in a search strategy. Trialstreamer is an AI‐based search tool from the makers of RobotReviewer. It automatically identifies all randomized controlled trials (RCTs) in Pubmed, uses text mining and normalisation to detect PICO entities, and makes results available via an intuitive search interface [9].

Search strategies developed for one electronic database (such as PubMed) cannot be simply copied and used in another database and systematic reviews should include several relevant databases to ensure searches are exhaustive. The Medline Transpose^9^ and Polyglot^10^ tools can help with the translation of search strategies designed for one database and translate them for use in another.

Searching for evidence should not be limited to indexed publications on electronic databases, but should endeavour to locate ‘grey’ literature, not formally published, while acknowledging its limitations. This helps to offset the difficulty of publication of negative results, which causes a bias within the published literature [10]. Additional supplementary searching can be supported by citationchaser,^11^ Paperfetcher,^12^ Scite.^13^ Publish or Perish also can retrieve and analyse citations but can also download Google Scholar search results which might be of value when undertaking supplementary searching.

## 5 Tools to Support Reference Management

Figure 1 shows that previous work identified 37 tools and software packages to assist reviewers and information specialists with deduplication, reference management, and citing [5]. Mendeley, as mentioned before, is one such tool. Generally, these tools are multi‐purpose and not specifically designed for systematic review. When using these tools in systematic review contexts to manage data, it is important to verify their reliability. Especially for deduplication, reviewers may consult published research that evaluates the performance of underlying algorithms to determine the amount of manual checking and review still needed [11].

## 6 Tools to Support Study Selection

Study selection is one of the most time costly elements of the review process, with search results often yielding very large datasets of potentially relevant reports to search through on abstract and subsequently full‐text level. This process requires not only the screening of reports itself, but also grouping multiple reports of the same study to avoid spurious results by counting study participants more than once. An analysis of 195 systematic reviews from registration to publication has shown a mean time of 67.3 weeks, although there existed a skew within that data set of reviews with large amounts of references (> 10,000) that may take significantly more time during the study selection phase [12]. Generally, recommended approaches suggest that two reviewers screen independently and then compare their differences, thereby reducing the risk of erroneously discarding relevant references, although single screening of references on abstract level may be acceptable as long as the final inclusion decision for a study (not report) is made in duplicate [13]. Evaluation of the methods of screening do confirm that when results are screened by only one reviewer, approximately 6% of eligible studies might be missed [14], but risks may be mitigated by the approaches discussed below. Of concern is also the risk of reviewer bias, with either a conscious or unconscious bias influencing the selection of the data set.

Tools to support the process of study selection can do so in the following ways:

1. Tools that enable the process of independent screening to be managed in such a way that allows ready checking and reconciliation processes. For example, a screening tool may support a pilot screening phase where a set number of references, for example 100, are screened by all reviewers in the project. Subsequently, the reviewers resolve any conflicts as a group to clarify open questions or misunderstandings.
2. Tools that use active machine learning approaches to streamline identification those references that are most likely to meet the inclusion criteria. Active learning means that the tool re‐orders likely relevant references to the top of the screening pile whenever the reviewers identify an included reference during screening. Those references that are predicted to be the most relevant are prioritised and thus identified more rapidly at the beginning of the screening process. Later during the screening process, reviewers may then make the decision to switch to single screening for the remainder of the project, knowing that most of the likely relevant references have already been screened in duplicate.
3. Tools that implement statistical early‐stopping algorithms. Active learning, as described in the previous paragraph, may help reviewers to expedite the identification of references but it cannot provide a data‐driven estimation of the proportion of relevant references that have been identified at any point during the live screening process. It thus makes it unsafe, and may be considered not methodologically rigorous, to stop screening early to save time when only relying on reference prioritisation algorithms. However, in recent years, a number of statistical algorithms have been proposed to estimate the likelihood of 95%, 99%, or 100% identification of relevant references during a live screening process [15, 16, 17, 18, 19]. In the evaluation of one tool, the screening‐burden was estimated to be reduced by up to 60% [16], but bigger evaluation datasets and fair comparisons between algorithms are likely to improve methods and the adoption into digital evidence synthesis tools [20]. However, reviewers are not forced to stop early—they may well elect to use the early‐stopping threshold to make an informed decision to switch from dual to single‐screening.

Given the heavy time burden that study selection (screening) can take, it is not surprising that machine learning support tools have seen the greatest development, or evaluation. As of December 2024, the Systematic Review Toolbox website [6] (see Figure 1) that gives an overview of digital evidence synthesis tools, records 45 study selection tools for systematic reviews (although some of these may no longer be maintained).

A risk is that these approaches learn by identifying papers closely aligned to those already selected. In some types of reviews, for example in reviews undertaking broad research questions, this may lead to some references being systematically missed. For example, if the research objective is to identify the range of types of interventions reducing the risk of preterm birth, the need to look at a breadth of types of intervention might not be best served by an approach that prioritizes those that are most like the ones already included.

Lastly, automation tools for reference priorisation and early stopping need to be evaluated on the basis of a sufficiently large data set of already completed systematic reviews as gold‐standard. This encompasses including large and small reviews, as well as reviews with narrow and very broad inclusion criteria. Ideally, evaluations should use publicly available gold‐standard datasets to increase comparability with other approaches for initial publication. Before choosing a method, reviewers should inform themselves if any truly independent evaluations from research teams not related to the chosen tool have been published.

In Table 2, potential tools to support screening are given, with their functions, cost, and also some references that have evaluated their benefit to the review process in terms of time saved and accuracy.

**Table 2:** Tools to support study selection.

Tools	Functionality	Cost	Evidence of benefit/risks
Abstrackr	Priority screening	Free, open‐source code	By product owner [21]
			Independent [22, 23, 24, 25, 26, 27, 28, 29, 30, 31]
EPPI reviewer	Priority screening, studification	Subscription, open‐source code	By product owner: website^a^
			Independent [32]
Distiller	Priority screening	Subscription	By product owner: blog article^b^
			Independent [23, 33, 34, 35]
SWIFT‐ActiveScreener	Priority screening, early‐stopping	Subscription	By product owner [16, 36]
			Independent [37]
Covidence	Priority screening, studification	Subscription	By product owner: website^c^

*Note:* Common automation functionalities are priority screening, where references are re‐ordered during the screening process to present likely relevant includes first, commonly using machine‐learning. Studification is a workflow task of identifying and grouping multiple reports of the same study to ensure that data for each participant is included only once in the analysis, this task remains manual but not all tools possess built‐in infrastructure to support it. Early‐stopping refers to an additional statistical algorithm that estimates when reviewers can stop the priority screening process early to save workload.

^a^ https://eppi.ioe.ac.uk/CMS/Portals/35/machine_learning_in_eppi-reviewer_v_7_web_version.pdf (last accessed 22/04/2025).

^b^ https://www.distillersr.com/products/distillersr-systematic-review-software (last accessed 26/06/2024).

^c^ https://www.covidence.org/ (last accessed 26/06/2024).

## 7 Tools to Support Data Extraction

Data extraction (sometimes called coding) ensures that included papers are approached in a standardised manner, with data extracted that allows direct comparison and analysis between studies. It requires the considered and tested development of a data extraction form or spreadsheet and the data extracted from each included study will be determined by the research objectives. The data will include details of the study such as identifiers, setting and year of publication. It is also likely to include details of the included population, the study design, and outcomes of interest. The process of data extraction may also include (for most types of evidence synthesis) the critical appraisal of the included studies and can be preformed in parallel to data extraction.

Tools to automate the process of data extraction have had less development and evaluation. While a recent living review of automated data extraction found 76 publications on the topic, only 6 described implemented end‐user tools and an even smaller number of these tools are available and usable. The remaining published algorithms looked at extracting more than 30 different types of data from text, but 84% focused on doing so only from titles and abstracts. This decreases their utility in real‐world evidence synthesis projects because abstracts are still likely to miss important information [38]. Of the usable tools identified in that review, two are mining title and abstracts to provide a PICO search tool: Trialstreamer is a free open‐source tool described in the previous section, and Trip database^14^ is a tool with some free and some paid premium functionalities, covering a similar use‐case [39]. The other three tools, RobotReviewer, Exact, and ACTA are contained in Table 3 below. Some tools have been developed to support different elements of the data extraction process. For example, WebPlotDigitizer can support the extraction of data from graphs and other figures.^15^

**Table 3:** A selection of tools available to support data extraction from papers for subsequent analysis.

Tool	Action	Cost	Evidence of benefit/risks
OpenMeta	Support outcome data for meta‐analysis	Free	[40]
RobotReviewer	Automates the risk of bias assessment and data extraction in RCTs	Free, open‐source code	[41, 42, 43, 44, 45, 46, 47, 48, 49]
ExaCT [50]	Extracts study characteristics from the full‐texts of RCTs.	Free	[50]
ACTA [51]	Natural Language Processing (NLP) and Machine Learning method to extract data from trials.	Free, open‐source code	[51]

A number of tools are available that support the extraction of outcome data (Table 3) OpenMeta[Analyst],^16^ for example, supports the extraction of numerical data for meta‐analysis.

## 8 Tools to Support Critical Appraisal

Critical appraisal is also a time‐consuming element during the data extraction process. Human time taken was measured at approximately 10 min per outcome by one study [52]. This data comes from assessments done with the first Cochrane Risk of Bias tool (RoB1), which is also the tool version for which assessments are automated by the digital tools discussed in this section. Other, or newer, risk of bias assessment methods may take longer to assess. Reviewer experience may be another factor prolonging the time taken during this review step, as is the complexity or opaqueness of reporting within the paper that is being assessed.

Numerous paper‐based checklists have been created to support the process of critical appraisal, with the Systematic Review Toolbox website alone listing 78 such guidance, checklists, or appraisal tools for systematic reviews in June 2024 [6].

Some digital tools have been developed which also support the extraction of data that is needed to support the critical appraisal. RobotReviewer is one that can support the extraction of risk of bias elements in randomised controlled trials (RoB 1). It aims to mitigate the time burden of data extraction by (semi‐) automating some elements of the data extraction process using machine learning and natural language processing and has been evaluated by the tool developers as well as by independent users (see Table 3). Another currently functional web tool to automate risk of bias assessments (RoB1) was developed by Millard et al., [53]. Currently, we are not aware of any digital tools with the capability of assessing the risk of bias with Cochrane's latest RoB2 tool. However, a recent methods paper has shown the feasibility of using Large Language Models to automate assessments across 10 bias domains using a modified RoB2 version [54]. We are also not aware of any automation tools addressing bias assessment using frameworks other than RoB, with digital tools essentially limited to customised Excel spreadsheets [55].

## 9 Tools to Support Creation of Visual Presentation of Findings

Visualisation of findings can greatly aid the communication of review findings. Forest plots are an example of the visual outputs that can support the understanding and interpretation of the review findings. Many types of evidence synthesis do not undertake a project of statistical summation and other approaches to the visual presentation of findings my support their presentation.

Examples include the use of heatmaps to show distribution of evidence. The use of Evidence and Gap Maps which provide visual but also interactive outputs are examples of innovations that can help knowledge users engage with the results of a review.

**Figure 2:**
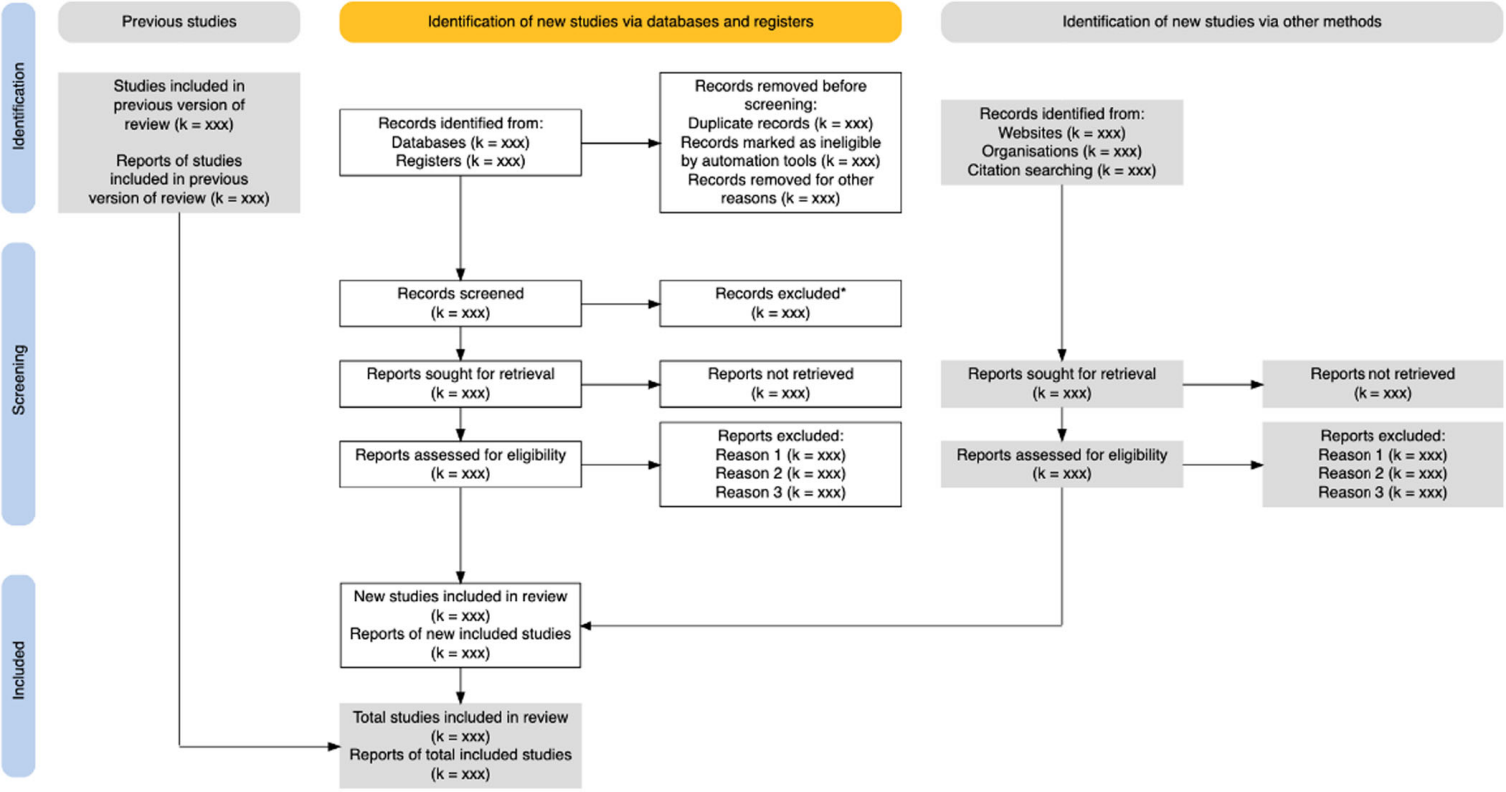
An example of a PRISMA diagram showing how references are found, assessed for relevance and included or excluded in the final analysis. *Source:* https://www.eshackathon.org/software/PRISMA2020.html.

Tools also exist to support the production of the PRISMA flow diagram (see Figure 1) and the quality appraisal diagram if using ROB (see Figure 2)—creating visuals such as the ones below and saving researchers a considerable amount of time and helps to communicate methodology and findings clearly.

### 9.1 The Use of LLM and Future Developments in Use of AI‐Enabled Tools

There is an increasing number of generative language models and tools for data analysis and text creation, some of which are freely available online, for example ChatGPT 3.5, Petal, Perplexity, Elicit to mention a few; while some other ones are behind the paywall, for example Scite. The commonality is that they work best with scaffolding, a technique consisting of providing lots of examples as prompts which the model can learn from, for example multi‐PDFs chat. This is where the tagging previously conducted at screening can be useful as a foundation of your writing. Multiple‐PDFs chat is only available in the ‘Advanced Data Analysis’ section of ChatGTP 4 (the paid version of ChatGTP) but the free version of Petal can give you a glimpse of multiple documents interrogation.

Although it may be tempting to ask these tools to do the job for you, it might be worth reiterating that these tools cannot be fully trusted and close scrutiny needs always being exercised.

### 9.2 Data Security and Tool Availability

Any review team adopting a tool for their review should ensure that data are secure and stored in a way that complies with their institution's requirements regarding data protection and privacy. For example in the European Union (EU), the General Data Protection Regulation (GDPR) stipulates that data stays private and that tool developers may not access it to further refine commercial in‐house AI algorithms unless the user explicitly consents.^17^ Tool providers with servers outside the EU might not adhere to GDPR principles, and users should always collect some basic information on who owns a tool and which local consumer protection laws apply. Furthermore, reviewers should consider the implications of web‐based tools suddenly no longer being maintained by their developers. A very simple security measure to avoid being impacted by cyber‐threats and surprising de‐commissioning of tools is to download and back up data at regular intervals.

## 10 Conclusion

Systematic review methodology to underpin decision making has had less impact within the field of pathology than it has elsewhere, particularly in histopathology. The central role of the randomised controlled trial in evidence‐based medicine and the perceived complexity of performing systematic review may in part explain this reluctance. RCT's while valuable to exploring what works for treatment, are not the gold standard approach for the diagnosis of disease.

Other types of evidence are arguably of greater value in pathology, and the types of questions that are being asked differ to those addressed by an RCT design. One of the limitations of many of the tools developed to date to make systematic review more efficient is their focus on RCTs. However, tools for screening and managing the process of the review have relevance for reviews incorporating a wide range of study designs and can be helpful in any discipline, including pathology. As an example of what can be done in pathology, the EVI MAP project [56], is mapping all existing evidence underpinning tumour classification, and will support the forthcoming 6th edition of the WHO Classification of Tumours, to reduce bias and improve the evidence used to determine the characteristics of each tumour type [57].

**Figure 3:**
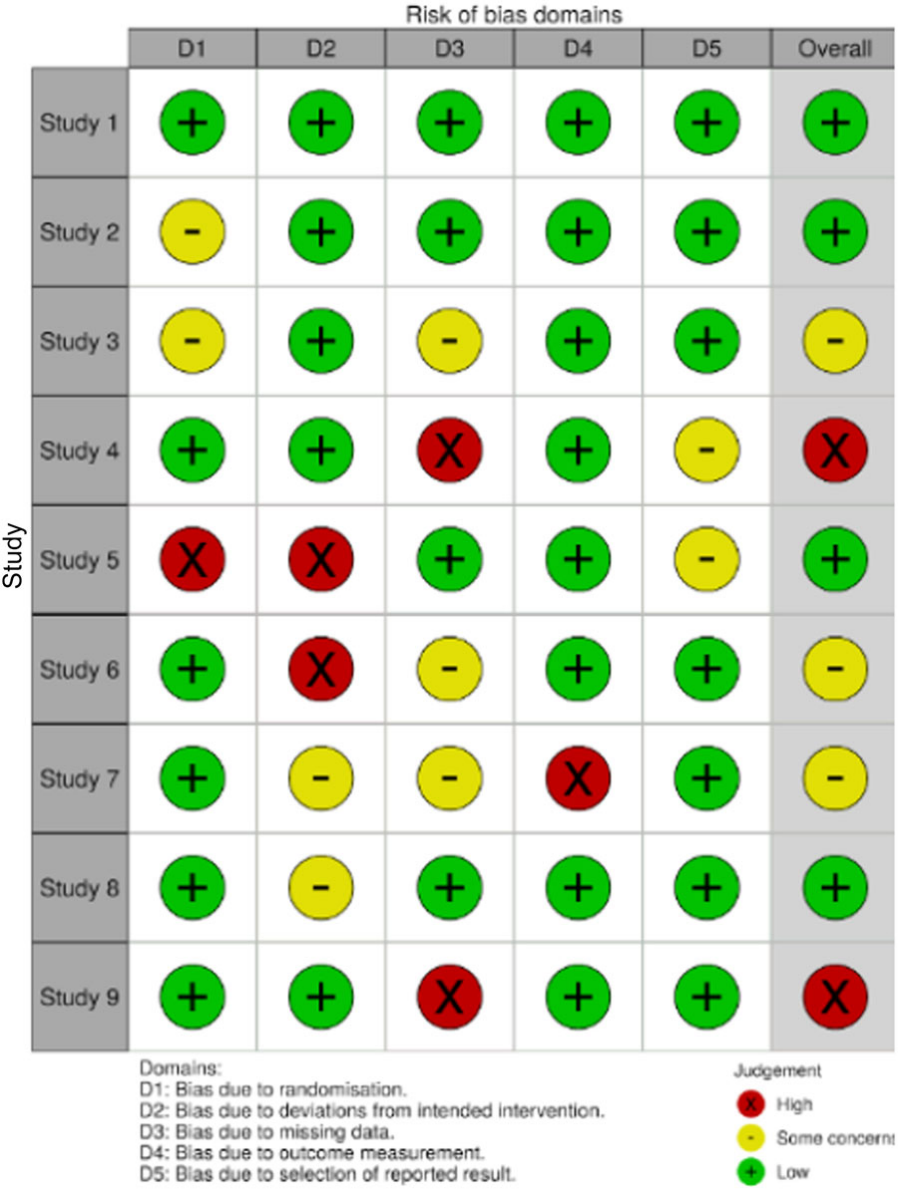
An example of a risk of bias tool used for the assessement of bias in individual studies under review. *Source*: https://www.riskofbias.info/welcome/robvis-visualization-tool .

Evidence based methods have evolved to support a wide range of research questions, including those relating to epidemiology, diagnosis, prognosis and barriers to treatment. Evidence based practice makes sense of the science, ensuring that clinical care is not simply based on opinion, but on the judicious combination of rigorous evidence, clinical expertise and patient preferences. In an era where the growth of paper mills and fraudulent research is targeting the field of cellular biology of cancer, the need for rigorous assessment of published evidence in a timely manner has never been more pressing. The process of producing rigorous evidence synthesis is time consuming, costly but critical. Tools can help to support the review process, reducing the time needed but maintaining rigour. Transparency in methods should remain at the forefront and the tools used referenced, and how they are used described and reported. The learning curve needed before users can competently employ a tool often acts as a barrier to the uptake of available tools in evidence synthesis, particularly for those who might be new to the methodology. So, before starting, it is important to take time to become familiar with the options and their possibilities. No tool can or should replace the human input needed to synthesise and interpret the results. While tools can help, they are only ‘a tool’ and as such are there only to be useful, but not to replace the expert. (Figure 3).

## Disclosure

The content of this article represents the personal views of the authors and does not represent the views of the authors’ employers and associated institutions. Where authors are identified as personnel of the International Agency for Research on Cancer/World Health Organization, the authors alone are responsible for the views expressed in this article and they do not necessarily represent the decisions, policy or views of the International Agency for Research on Cancer/World Health Organization.

## Ethics Statement

The authors have nothing to report.

## Consent

The authors have nothing to report.

## Conflicts of Interest

The authors declare no conflicts of interest.

## Data Availability

Not applicable, all data are available within the manuscript.

## Endnotes

1 See https://www.campbellcollaboration.org/2023/11/stepping-up-evidence-synthesis/, https://training.cochrane.org/resource/artificial-intelligence-technologies-in-cochrane, https://sumari.jbi.global/ (last accessed 28/12/2024).

2 See https://training.cochrane.org/handbook (last accessed 26/06/2024).

3 See https://www.mendeley.com/ (last accessed 26/06/2024).

4 See https://systematicreviewtools.com/ (last accessed 28/12/2024).

5 See https://hgserver2.amc.nl/cgi-bin/miner/miner2.cgi (last accessed 26/06/2024).

6 See https://mesh.med.yale.edu/ (last accessed 26/06/2024).

7 See https://pypi.org/project/wordfreq/ (last accessed 26/06/2024).

8 See https://elizagrames.github.io/litsearchr/ (last accessed 26/06/2024).

9 See https://medlinetranspose.github.io/ (last accessed 26/06/2024).

10 See https://sr-accelerator.com/#/polyglot (last accessed 26/06/2024).

11 See https://www.eshackathon.org/software/citationchaser.html (last accessed 26/06/2024).

12 See https://paperfetcher.github.io/ (last accessed 26/06/2024).

13 See https://scite.ai/home (last accessed 26/06/2024).

14 See Trip Medical Database (tripdatabase.com) (last accessed 26/06/2024).

15 See https://automeris.io/WebPlotDigitizer.html (last accessed 26/06/2024).

16 See http://www.cebm.brown.edu/openmeta/ (last accessed 26/06/2024).

17 See https://europa.eu/youreurope/citizens/consumers/internet-telecoms/data-protection-online-privacy/index_en.htm (last accessed 28/12/2024).
